# The effect of a multi-component intervention on disrespect and abuse during childbirth in Kenya

**DOI:** 10.1186/s12884-015-0645-6

**Published:** 2015-09-22

**Authors:** Timothy Abuya, Charity Ndwiga, Julie Ritter, Lucy Kanya, Ben Bellows, Nancy Binkin, Charlotte E. Warren

**Affiliations:** Population Council, PO Box 17643-00500 Nairobi, Kenya; School of Medicine, University of California San Diego, La Jolla, CA USA; Health Economics Research Group, College of Health & Life Sciences, Brunel University London, Uxbridge, UB8 3PH, London, UK; Population Council, Plot 3670, No. 4 Mwaleshi Road, Olympia Park, Lusaka, 10101 Zambia; Population Council, 4301 Connecticut Ave NW, Suite 280, Washington, DC 20008 USA

## Abstract

**Background:**

Disrespect and abuse (D & A) during labor and delivery are important issues correlated with human rights, equity, and public health that also affect women’s decisions to deliver in facilities, which provide appropriate management of maternal and neonatal complications. Little is known about interventions aimed at lowering the frequency of disrespectful and abusive behaviors.

**Methods:**

Between 2011 and 2014, a pre-and-post study measured D & A levels in a three-tiered intervention at 13 facilities in Kenya under the *Heshima* project. The intervention involved working with policymakers to encourage greater focus on D & A, training providers on respectful maternity care, and strengthening linkages between the facility and community for accountability and governance. At participating facilities, postpartum women were approached at discharge and asked to participate in the study; those who consented were administered a questionnaire on D & A in general as well as six typologies, including physical and verbal abuse, violations of confidentiality and privacy, detainment for non-payment, and abandonment. Observation of provider-patient interaction during labor was also conducted in the same facilities. In both exit interview and observational studies, multivariate analyses of risk factors for D & A controlled for differences in socio-demographic and facility characteristics between baseline and endline surveys.

**Results:**

Overall D & A decreased from 20–13 % (*p* < 0.004) and among four of the six typologies D & A decreased from 40–50 %. Night shift deliveries were associated with greater verbal and physical abuse. Patient and infant detainment declined dramatically from 8.0–0.8 %, though this was partially attributable to the 2013 national free delivery care policy.

**Conclusion:**

Although a number of contextual factors may have influenced these findings, the magnitude and consistency of the observed decreases suggest that the multi-component intervention may have the potential to reduce the frequency of D & A. Greater efforts are needed to develop stronger evaluation methods for assessing D & A in other settings.

## Background

Pregnancy and childbirth are important rites of passage, with deep personal and cultural significance for women and their families, bringing joy along with great physical and emotional vulnerability. Most international efforts for strengthening maternal health care focus largely on preventing morbidity and mortality, but recent emphasis on the universal right of quality maternal health care has illuminated the need for examining interpersonal relationships between patients and providers and, more specifically, ensuring women give birth in respectful circumstances and are not subjected to disrespectful or abusive care.

The emphasis on the rights of both patients and health workers has its foundation in the Universal Declaration of Human Rights. Other international covenants such as the economic, social and cultural rights focuses on the right to everyone enjoying the highest attainable standard of physical and mental health [1]. More recently, protection of human rights for maternal and newborn issues has been emphasized. The Human Rights Council has, for example, called upon States to scale up efforts to achieve the integrated management of quality maternal, newborn and child health services, particularly at the community and family levels, and technical guidance documents on a human rights based approach to reduce maternal morbidity and mortality [2] and mortality and morbidity of children under 5 years of age [3] have been published. Such an approach can help improve quality care at birth both for the mother and the newborn. Treating women with dignity and respect is central to improvements of quality of care.

Not only is disrespect and abuse (D & A) a human rights issue, but it has been recognized as an equity issue, as well as a public health concern, with recent attempts to better define it [4] as well as describing and measuring its occurrence [5, 6]. Studies from Tanzania and Kenya have demonstrated that D & A is a common phenomenon; in these studies, its frequency among health facility maternity clients ranged from 20–28 %, depending on study and interview locations [6, 7]. These high rates of D & A, along with its contributions to continued home deliveries with no skilled attendance [5, 8–11]; provoke questions about what interventions can decrease it.

Although facility-based experiences of D & A during childbirth can be viewed as a quality of care issue, examining D & A through a quality of care lens alone will likely neglect potential interventions beyond clinical processes and structural features. Reducing D & A requires much broader societal contribution, at both policy and community levels. Using both a human rights lens and a quality of care lens to examine and implement interventions is likely to improve provider attitudes and ensure that both patient and provider rights are respected.

In 2011, the *Heshima* project in Kenya launched complementary interventions at community-level, facility-level, and policy-level. In addition, the Heshima project undertook quantitative and qualitative assessments to test associations between the implementation of D & A activities and trends in quality of care at intervention facilities. In this paper, we present the quantitative findings of *Heshima*’s impact on perceived and observed D & A behaviors among women delivering in participating facilities, building upon earlier work by the project published elsewhere [4, 10, 12, 13].

### Description of the intervention and its context

In 2010, the year before *Heshima*’s implementation, the Kenya Demographic and Health Survey (KDHS) reported Kenya’s maternal mortality ratio at an estimated 488 deaths per 100,000 live births, with only 43 % of births occurring in health facilities in the previous five years [14]. In Kenya at the time, there was growing policymaker awareness of the need to reduce maternal mortality [15, 16], along with front page newspaper stories exposing health facilities’ detainments of infants and their mothers for unpaid hospital fees, which provoked national concern over the quality of services.

In response to these concerns, the *Heshima* project developed simultaneous policy, facility and community-level interventions to mitigate D & A. Initiated in June 2011 and completed in February 2014, the project was implemented in 13 facilities in Kisumu, Kiambu, Nyandarua and Uasin Gishu counties as well as in a slum in the outskirts of Nairobi. *Heshima* overlapped with a larger evaluation of the Kenya reproductive health voucher program and involved facilities accredited as maternal health voucher facilities (in Kisumu and Kiambu) as well as comparable facilities in counties (Nyandarua and Uasin Gishu) with no voucher programs [17], in both rural and urban settings.

*Heshima* utilized an iterative process of learning-by-doing throughout its design, development and assessment, with the objective to identify low-cost and feasible policy, facility and community interventions. Details of the project design have been previously published [13]. Facility interventions, beginning in six facilities for 20 months, were refined and replicated in seven additional facilities beginning in November 2012, and continued for 14 months. The interventions were continuously reviewed through stakeholder consultations. Key intervention activities in facilities and communities are described in Table 1.

**Table 1:** Key components of facility and community-level interventions

Intervention Activity	Implementation activities
Facility level	
Training in promoting respectful care including values clarification and attitude transformation (VCAT)	Three day training on VCAT based on providers’ and clients’ rights and obligations. Revision of professional ethics and practices. Each of the study facilities developed action plans for institutionalization in maternity units.
Quality Improvements teams (QITs)	Strengthen facility quality improvement teams (e.g. health facility management committees-HFMCs) for monitoring, addressing, and resolving D & A cases and address infrastructure, drugs and commodity supply concerns. Additionally HFMCs were trained on rights and obligations related to childbirth, develop D & A protocol for reporting and monitoring, and encouraged community membership.
Caring for Carers	Counseling for providers at the group and/or individual levels to support providers with coping mechanisms to overcome experiences related to high workload, trauma or critical incidents. Initially this was conducted by FIDA counselors (one counseling session per site) while at the same time they would role model the sessions for trained counselor with the facility or within reach of the county if the facility does not have any. This site level counselor would then continue with counseling session in their respective sites.
D & A Monitoring	Providing mechanisms to report D & A such as customer service desks, suggestion boxes and through consortium supervision visits by implementing team. Also the county health teams and facility quality improvements conducted monitoring as part of routine work.
Mentorship	On-the-job role-modeling for provider behavior change by identified champions within the facility as part of routine continuous professional education.
Maternity Open Days	Trust-building with local communities during which men and women from the community can visit the nearby facility and learn about procedures in the maternity wards and interact with nurse-midwives.
Community level	
Community workshops	Civic education of community rights to sexual and reproductive health including maternal health care by community health (extension) workers (CHWs and CHEWs) associated with a particular county area and facility conducted by the partners but by led by Federation of women Lawyer-Kenya. Trainers (CHWs, CHEWs, opinion leaders, civil and legal aids) conducted respectful care sensitization meetings for community members (women, men and youth) with support of their respective county health management teams. Deliberate effort was made to involve male in community workshops as participants and facilitators as well through targeted meetings for men ‘calling them to action’ to demand respectful care for their wives and partners.
Mediation/alternative dispute resolution	Training society leaders (e.g. CHWs, respected persons) on mediation skills, to act as intermediaries between community members and the health facility to address D & A issues. Mediators were selected by communities and facilities (on set criteria) and trained by Federation of women Lawyer-Kenya
Counseling Community Members	Counseling community members who have experienced D & A by Federation of women Lawyer-Kenya and other professional counselors within the facilities. These would be referral from CHWs or community legal aids.

In brief, the process entailed an interactive three-tiered set of interventions at policy, facility and community levels. At the policy level, continuous policy dialogue took place in technical meetings with government, civil society and professional knowledge networks served as a way to build rapport and ownership as well as compelling critical actors to reflect on D & A as a key component of quality of maternity care. At the facility level, the core intervention elements included the orientation and training on respectful maternity care for providers and managers. The orientation aimed to improve providers’ attitudes and their working environment and to strengthen linkages between the facility and community for accountability and governance. At the community level, the core Heshima interventions included training on respectful maternity care issues, community dialogue, and counselling. In addition, it included a mechanism whereby reported cases of D & A would be examined by a mutually agreed-upon mediator who facilitated a resolution between the woman reporting the D & A and the facility providers and management.

During the implementation period, several contextual influences were critical to the intervention outcomes. For example, ongoing devolution of Kenya’s health services, which began in June 2013, created new organizational structures and processes that led to significant structural changes in human resources for healthcare. A second major development was the mid-2013 mandate of free maternity service provision in all public health facilities. Two nursing strikes affected service delivery as well and slowed implementation in study facilities, with the first strike, of two months, beginning in December 2012, and the second beginning in December 2013 and lasting for three months.

## Methods

### Study design

Our study used a before-and-after design, without a comparison group, to measure the effect of interventions to reduce the prevalence of D & A during labor and delivery in 13 Kenyan health facilities. Baseline data were collected between September 2011 and February 2012, with endline data collected in January and February 2014. The study conducted two data collection exercises: exit interviews with women who had just delivered and observation of women, from their early labor to post-delivery, conducted by trained nurses and midwives.

### Study setting

The 13 purposefully selected facilities constituted different facility types (public, private, faith-based) and different levels of care, comprised of three public referral hospitals, three district (public) hospitals with maternity units, two faith-based hospitals, two private nursing homes, and one (public) health center. Four of the 13 facilities were rural and the rest were in urban or peri-urban areas. Facilities in the study employed 58 specialist doctors, 116 medical doctors, 1,503 nurses or midwives, 27 theater nurses, 48 anesthetists, and 126 pharmacists. The 13 facilities, combined, had 21 delivery couches and a total bed capacity of 194 in the labor wards. Outpatient health facilities (heath centers or clinics) had only one nurse or midwife per shift, while larger ones (hospitals) employed nine to 11 per shift.

### Enrollment and data collection procedures

#### Client exit interviews

All women 15–45 years old who had delivered within 24–48 hours in a participating facility were eligible for inclusion, regardless of pregnancy outcome. *Heshima* researchers approached all postpartum women discharged from the postnatal ward, described the study and its interview process, emphasizing its privacy and confidentiality, and their consent to participate was requested, utilizing a structured consent form in the woman’s preferred language. Women were recruited until the necessary sample sizes were reached for all 13 study facilities [13]. During the September 2011 through January 2012 baseline period, a total of 641 women consented to participate; during the January and February 2014 endline, 728 women consented to participate. Fifty percent (50 %) of all women who delivered in the facilities in the previous 48 hours participated in the study’s baseline survey, and 60 % participated in the endline survey.

Interviews were conducted in a specially designated room at each facility by interviewers trained in the study procedures to ensure that patient privacy was maintained. The questionnaire includes modules that examine women’s demographic and household characteristics including their socio-economic status, past service utilization, characteristics of their deliveries, their perceived quality and satisfaction, and experiences of D & A. Table 2 presents the questions used to assess D & A experiences. Portable digital assistant (PDA) devices were used to collect the data, which were downloaded into an MS Access database before their export to Stata 11 for data management.

**Table 2:** Questions for assessment and corresponding categories

Study methods and examples of Questions used	Corresponding categories
Client exit survey	
Were you physically abused by any of the health care workers	Physical abuse
Were you treated in a way that violated your privacy?	Privacy violation
Were you treated in a way that violated your confidentiality	Confidentiality violated
Did any healthcare provider talk or use a tone or facial expression that made feel uncomfortable?	Verbal abuse
Were you or your baby prevented from leaving this facility because you could not pay	Detainment
Were you left un attended by health providers when you needed care	Abandonment
Observations of provider-patient interactions	
Examination	
Provider did not obtain permission from the mother before the initial examination or did not seek the mother’s consent for the vaginal examination	Non-consented care
When the provider did not use “dignified language” or using “harsh tones or shouting” during the history taking	Verbal abuse
When either there were no separating partitions between the beds or the partitions didn’t provide privacy	Lack of privacy
Delivery period	
Assessed as the staff being aggressive in any way and the midwife/nurse research assistant indicating whether it was physical aggression or verbal aggression (or both)	Physical aggression
	Verbal aggression
Assessed as the mother not being covered while being moved from pre-labor ward to the delivery room or not having closed partitions or being uncovered (excluding the perineal area).	Lack of privacy
Postnatal period	
Was the mother not having a bed allocated only to herself	Bed sharing

#### Observations of provider-patient interaction during labor and delivery

For the observations of provider-patient interaction, participants were in early labor, ages 15–45, who provided their informed consent for observation of their labor and delivery, with key actions recorded. In each study location, a trained researcher approached the facility’s patients in early labor as they entered the maternity unit, explained the study and its objectives, and requested their written consent for the observation. The structured, non-participant observations in maternity units measured both process (how patients are treated) and content (what they were told, revealing technical competency, accuracy of information and provision of essential information) of services. Data were collected on paper questionnaires, keyed into EpiData 3.1, and exported to Stata 11 for cleaning and analysis.

Researchers were nurses or midwives trained to conduct the observations. A list of potential situations requiring their possible intervention (e.g., emergencies such as heavy vaginal bleeding if staff was otherwise not available) was developed by the Ministry of Health, and if such life-threatening situations occurred, they were allowed to provide immediate emergency care. For all such situations, the observation was terminated and was not included in the analysis.

### Data analysis

Final analyses of both the exit interviews and observation of provider-patient interaction utilized SAS software, Version 9.4 (Cary, NC, USA).

#### Client exit interviews

To measure the intervention’s effects in reducing the occurrences of D & A, a Likert scale operationalized an accepted definition of D & A [4]. The key outcome of interest was the percentage of women responding with any answer other than 5 to the question “On a scale of 1 to 5, were you treated in a way that made you feel humiliated or disrespected? *1 means very humiliated and 5 means not humiliated*.” The study also sought to examine the intervention’s effects on six categories of D & A including occurrence of physical abuse, violation of privacy as well as confidentiality, verbal abuse, detainment, and abandonment [6]. To assess these categories of D & A, women were asked to provide a “yes” or “no” response to the questions listed in Table 2.

A wealth indicator variable was created to represent socio-economic status based on questions assessing a woman’s household ownership of specific items (radio, television, bicycle, phone, refrigerator, scooter, automobile) as well as household characteristics (flooring and roofing materials, water sources, toilet facilities, electricity). Principal component analysis generated factor scores, and an overall asset score was calculated for each participant. Wealth terciles were constructed using the final asset scores, which were based on an analysis of the whole sample, including baseline and endline participants, to control for socio-economic differences between the two groups.

Bivariate analyses utilized a chi-square test to determine if baseline and endline participants were significantly different in their socio-demographic and delivery characteristics. Unadjusted and multivariate logistic generalized linear mixed models (GLMM), with the facility as a random effect and all other variables as fixed effects, assessed differences in D & A for baseline and endline participants, as well as the association of D & A with other characteristics. Covariates included in all multivariate models included woman’s age, parity, socio-economic status, time of delivery, marital status, accompaniment by another adult, and facility type. Other covariates considered for inclusion in models included education, any lifetime experience of physical abuse or rape, whether referred or presented at the facility directly, and primary service provider. All of these additional covariates were evaluated in each model and preserved only if statistically significant (*p* < 0.05), or if their presence altered the magnitude of association between time (baseline/endline) and D & A outcome by at least 10 percent.

#### Client-provider interactions observations

Seven indicators of the categories of D & A were selected, with matching measures at baseline and endline (Table 2), with three indicators for initial examination (non-consensual care, verbal abuse, lack of privacy), three during delivery (physical aggression, verbal aggression, lack of privacy), and one for postpartum care (bed sharing). Covariates included age, parity, time of delivery, facility type, and voucher status.

Analyses of patient and provider observations followed a methodology similar to the patient exit interviews. Bivariate analyses assessed the characteristics of the sample at baseline and endline, while unadjusted and multivariate logistic GLMM assessed differences between baseline and endline for D & A and it’s socio-demographic and facility risk factors. As with other analyses, the facility was included as a random effect. Covariates included all five of the previously enumerated exposure variables.

## Ethical considerations

The research protocol was approved by the Division of Reproductive Health of Kenya’s Ministry of Public Health and Sanitation, as well as the Kenya Medical Research Institute (KEMRI)’s Ethical Review Board (SCC 288) and Population Council’s Institutional Review Board (Protocol 517).

## Results

### Characteristics of interviewed postnatal women

As shown in Table 3, in both the baseline and endline groups, mean age was approximately 25 years, and most women were married or cohabiting, and almost all were either Protestant or Catholic. Approximately 40 % of women in both groups had not given birth before, and relatively few had delivered three or more times (13.1 % at baseline and 11.8 % at endline). Most patients had completed primary or secondary levels of education, although the endline group had a generally higher education level than the baseline group (*p* < 0.0001). Wealth distribution also differed among the two surveys, with more women in the post-intervention survey in the highest wealth tercile (41 % versus 25 %) and, proportionately, fewer in the lowest tercile (24 % versus 44 %) (*p* < 0.0001). Reports of any past physical abuse were high (19 %) at baseline, and even higher in the post-intervention population (38 %), a difference that is statistically significant (*p* < 0.0001). Although the rates were considerably lower for reported rape, significantly more women reported ever having been raped in the endline survey (4.8 %) that at baseline (2.2 %).

**Table 3:** Socio-demographic and delivery characteristics of maternity patients participating in baseline (2012) and endline (2014) surveys of the Heshima project in 13 facilities in Kenya, *N* = 1,369*

	Baseline (*N* = 641)	Endline (*N* = 728)	
	% (n)	% (n)	*p*-value^a^
Socio-demographic characteristics			
Age mean (SD)	25.0 (5.3)	25.2 (5.1)	0.473
Level of educational attainment			
No school	1.3 (8)	1.7 (12)	< .0001
Primary	46.3 (296)	33.9 (244)	
Secondary	42.6 (272)	47.9 (345)	
Tertiary/University	9.9 (63)	16.5 (119)	
Marital status			
Married/cohabitating	81.9 (525)	80.4 (585)	0.466
Not married	18.1 (116)	19.6 (143)	
Religion			
Muslim	1.3 (8)	2.6 (19)	
Catholic	25.8 (165)	26.2 (191)	0.309
Protestant	70.8 (453)	69.4 (505)	
Others/None	2.2 (14)	1.8 (13)	
Wealth quintile			
Poorest 33.3 %	44.0 (282)	23.9 (174)	
Middle 33.3 %	31.5 (202)	35.0 (255)	< .0001
Richest 33.3 %	24.5 (157)	41.1 (299)	
Previous births			
0	41.6 (266)	41.2 (300)	
1-2	45.3 (290)	47.0 (342)	0.710
3+	13.1 (84)	11.8 (86)	
Reported ever being physically abused	18.7 (120)	38.1 (277)	< .0001
Reported ever having been raped	2.2 (14)	4.8 (35)	0.009
Delivery Characteristics			
Facility sector			
Government/Council	91.0 (583)	88.1 (641)	0.082
Private/Faith based	9.1 (58)	12.0 (87)	
Voucher accredited facility	65.4 (419)	74.7 (544)	0.0002
Previous delivery at facility	25.7 (165)	27.2 (198)	0.542
Referred to facility	22.5 (144)	17.7 (129)	0.028
Time of delivery			
Day	57.4 (368)	53.2 (387)	0.115
Night	42.6 (273)	46.8 (341)	
Accompanied to facility	99.7 (639)	88.5 (644)	< .0001
Primary service provider for delivery			
Nurse/midwife	64.5 (404)	65.4 (474)	
Other	35.5 (222)	34.6 (251)	0.746

*Missing values < 5%

^a^Chi-square test of association

In both the baseline and endline surveys, almost all women surveyed were in government hospitals, and most delivered during the day, with two thirds attended by midwives or nurses. Significantly fewer women (18 %) at endline reported being referred from another facility for delivery compared to 23 % at baseline (*p* = 0.028). Accompaniment by another adult to the facility was higher at baseline than at the endline survey (*p* < 0.0001), but in both surveys the values exceeded 88 %.

#### Changes in reported prevalence of D & A

A 7 % absolute reduction in the prevalence of any feelings of humiliation or disrespect, from 20–13 % (odds ratio (OR) 0.6; 95 % CI: 0.4–0.8), was identified. The baseline and endline percentages of women reporting a primary outcome of humiliation or disrespect during labor and childbirth, along with the six individual typologies of D & A, are presented in Table 4. Unadjusted results for the six typologies showed that women surveyed at endline were significantly less likely to report physical abuse (OR 0.5; 95 % CI: 0.3–0.9), verbal abuse (OR 0.6; 95 % CI: 0.4–0.8), violations of confidentiality (OR 0.5; 95 % CI: 0.2–0.9), and detainment (OR 0.1; 95 % CI: 0.04–0.2) than women surveyed at baseline. Although not statistically significant, feelings of abandonment did increase, from 13 % at baseline to 17 % at endline (OR 1.3; 95 % CI: 0.9–1.8). Covariate adjustments resulted in greater effect sizes for physical abuse as well as violations of privacy, but with minimal changes for the other D & A categories (Table 4).

**Table 4:** Prevalence of reported disrespect and abuse during labor and delivery of maternity patients participating in baseline (2012) and endline (2014) surveys of the Heshima project in 13 facilities in Kenya, N = 1,369

	Baseline (*N* = 641)	Endline (*N* = 728)		
	% (n)	% (n)	OR (95 % CI)^a^	*p*-value^b^
Feeling humiliated or disrespected	20.1 (129)	13.2 (96)	0.58 (0.43 – 0.79)	0.0004
Physical abuse	4.2 (27)	2.1 (15)	0.47 (0.25 – 0.90)	0.024
Privacy violated	7.4 (47)	5.7 (41)	0.69 (0.44 – 1.08)	0.101
Confidentiality violated^c^	3.9 (25)	1.8 (13)	0.45 (0.23 – 0.89)	0.021
Verbal abuse	18.0 (115)	11.3 (82)	0.58 (0.42 – 0.80)	0.001
Detention	8.0 (51)	0.8 (6)	0.09 (0.04 – 0.22)	< .0001
Abandonment	12.7 (81)	16.9 (122)	1.28 (0.93 – 1.76)	0.124

^a^Endline vs. baseline; facility as a random effect to account for clustering

^b^Based on F test

^c^Due to a covariance estimate of zero, facility was not included as a random effect

#### Factors associated with reported D & A

Socio-demographic and facility characteristics associated with any form of D & A and its six typologies are listed in Table 5.

**Table 5:** Multivariate logistic GLMMs assessing risk factors for disrespect and abuse among maternity patients participating in baseline (2012) and endline (2014) surveys of the Heshima project, Kenya. Model includes time of data collection (baseline)*

	Any Humiliation or Disrespect	Physical Abuse	Verbal Abuse	Violation of Privacy	Violation of Confidentiality	Detainment	Abandonment
	AOR (95 % CI)	AOR (95 % CI)	AOR (95 % CI)	AOR (95 % CI)	AOR (95 % CI)	AOR (95 % CI)	AOR (95 % CI)
Time Endline	0.55 (0.40 – 0.75)^a^	0.38(0.17 – 0.82)^a^	0.57 (0.41 – 0.79)^a^	0.66 (0.41 – 1.05)^b^	0.33 (0.16 – 0.71)^a^	0.09 (0.04 – 0.23)^a^	1.25 (0.90 – 1.75)
Baseline	Ref	Ref	Ref	Ref	Ref	Ref	Ref
Age (for each additional year)	1.00 (0.96 – 1.03)	0.91 (0.84 – 0.99)^a^	1.00 (0.96 – 1.04)	1.03 (0.97 – 1.08)	1.03 (0.95 – 1.12)	1.00 (0.93 – 1.08)	1.01 (0.97 – 1.05)
Number previous births							
0	Ref	Ref	Ref	Ref	Ref	Ref ^a^	Ref
1-2	1.29 (0.89 – 1.88)	1.31 (0.59 – 2.90)	1.08 (0.73 – 1.61)	0.99 (0.57 – 1.74)	0.96 (0.41 – 2.27)	2.29 (1.02 – 5.14)	1.02 (0.69 – 1.51)
3+	1.03 (0.54 – 1.94)	2.28 (0.65 – 8.05)	1.31 (0.70 – 2.47)	0.85 (0.34 – 2.17)	0.64 (0.15 – 2.70)	3.51 (1.06 – 11.56)	1.02 (0.53 – 1.95)
Wealth							
Poorest 33.3 %	Ref	Ref ^a^	Ref	Ref	Ref	Ref ^a^	Ref ^b^
Middle 33.3 %	1.33 (0.89 – 1.99)	2.12 (0.95 – 4.71)	1.20 (0.79 – 1.81)	1.66 (0.89 – 3.10)	2.05 (0.86 – 4.94)	0.45 (0.21 – 0.96)	1.61 (1.04 – 2.49)
Richest 33.3 %	1.18 (0.76 – 1.83)	0.65 (0.22 – 1.88)	1.02 (0.64 – 1.61)	1.63 (0.82 – 3.23)	1.32 (0.48 – 3.63)	0.22 (0.08 – 0.60)	1.30 (0.80 – 2.09)
Time of Delivery							
Day	Ref ^a^	Ref ^a^	Ref ^a^	Ref	Ref	Ref	Ref
Night	1.37 (1.02 – 1.85)	2.51 (1.17 – 5.39)	1.47 (1.07 – 2.01)	0.96 (0.62 – 1.51)	1.40 (0.71 – 2.76)	1.15 (0.62 – 2.12)	1.14 (0.84 – 1.56)
Marital Status							
Married/cohabitating	Ref	Ref	Ref	Ref	Ref	Ref ^a^	Ref
Not married	1.28 (0.86 – 1.88)	1.29 (0.58 – 2.90)	1.12 (0.74 – 1.69)	0.81 (0.43 – 1.54)	1.37 (0.59 – 3.23)	6.65 (3.27 – 13.51)	1.12 (0.73 – 1.70)
Facility Sector							
Government	Ref ^a^	Ref	Ref	Ref	Ref	Ref	Ref
Private/FBO	0.37 (0.14 – 0.98)	1.07 (0.25 – 4.55)	0.47 (0.18 – 1.18)	0.58 (0.17 – 1.91)	0.79 (0.22 – 2.81)	0.23 (0.03 – 2.12)	0.52 (0.20 – 1.33)
Voucher Status							
No vouchers	Ref	Ref	Ref	Ref	Ref	Ref	Ref
Voucher accredited facility	1.36 (0.63 – 2.95)	1.88 (0.58 - 6.07)	1.26 (0.59 – 2.68)	1.42 (0.54 – 3.71)	0.86 (0.38 – 1.94)	1.42 (0.44 – 4.58)	1.36 (0.61 – 3.00)
Main Service Provider							
Doctor/Other	Ref	Ref	Ref	Ref	Ref	Ref	Ref
Nurse/Midwife	0.83 (0.60 – 1.13)	1.63 (0.77 – 3.47)	1.02 (0.73 – 1.43)	1.19 (0.73 – 1.94)	1.17 (0.56 – 2.45)	0.92 (0.48 – 1.77)	0.97 (0.70 – 1.35)
Referred to Facility							
No	**	**	**	**	**	Ref ^a^	**
Yes						4.31 (2.27 – 8.20)	
Physical abuse ever							
No	**	**	**	**	Ref ^a^	Ref ^a^	**
Yes					2.46 (1.19 – 5.06)	2.50 (1.30 – 4.82)	

*Missing values < 3 %

^a^Statistically significant at *p* < 0.05

^b^Statistically significant at *p* < 0.10

**Variable not included in the model

*Any D & A:* Delivering at night was associated with higher risk of D & A (adjusted odds ratio (AOR) 1.4; 95 % CI: 1.0–1.8), while delivering in a private or faith-based hospital was protective (AOR 0.4; 95 % CI: 0.01–0.98). No other socio-demographic or facility risk factors were statistically significant.

*Physical abuse:* Women in the middle wealth tercile had greater odds of reporting physical abuse than those in the poorest tercile (AOR 2.1; 95 % CI: 1.0–4.7), and women who delivered at night had 2.5 greater odds of reporting physical abuse than those delivering during the day (AOR 2.5; 95 % CI: 1.2–5.4). No other factors were significantly associated with this outcome.

*Verbal abuse:* The only factor significantly associated with verbal abuse was delivering at night (AOR 1.5; 95 % CI: 1.1–2.0).

*Violation of privacy and violation of confidentiality:* No socio-demographic or facility factors were significantly associated with either of these typologies.

*Detainment:* Women with one or two previous deliveries were twice more likely to be detained than women with no prior deliveries (AOR 2.3; 95 % CI: 1.02–5.1), and women with three or more previous births had an even higher risk (AOR 3.5; 95 % CI: 1.1–11.6). Marital status was also associated with detainment. Unmarried women had greater than six-fold higher odds (AOR 6.7; 95 % CI: 3.3–13.5). Women in the middle and upper wealth terciles were significantly less likely to be detained than women in the lowest tercile (AOR 0.5; 95 % CI: 0.2–0.96 and AOR 0.2; 95 % CI: 0.1–0.6, respectively). Patients referred to the facility where they delivered were more than four times more likely to be detained than those who came directly to a delivery facility (AOR: 4.3; 95 % CI: 2.3–8.2). Women who reported any prior physical abuse had a 2.5-fold higher risk of detainment (AOR 2.5; 9 % CI: 1.3–4.8).

*Abandonment:* Reported abandonment was significantly more common among women in the middle tercile than the poorest (AOR 1.6; 95 % CI: 1.04–2.5); abandonment was also higher for women in the highest tercile (AOR 1.3; 95 % CI: 0.8–2.1), although the difference was not statistically significant. No other demographic and facility factors were significantly associated with this outcome.

### Observed disrespect and abuse during labor, delivery, and the immediate postnatal period

#### Characteristics of women observed

The baseline and endline populations in the observation of provider-patient interaction were of similar age and parity (Table 6), and their distributions were similar to the patient exit interview populations. Significantly more observations of night shift deliveries (38 % versus 32 % daytime deliveries) occurred during the post-intervention period, and most observations were in government facilities.

**Table 6:** Socio-demographics and delivery characteristics of maternity patients whose care was observed as part of the Heshima project at baseline (2011) and endline (2014) in 13 facilities in Kenya, *N* = 1,200*

	Baseline (*N* = 677)	Endline (*N* = 523)	
	% (*n*)	% (*n*)	*p*-value^a^
Demographics			
Age			
Mean (SD)	24.5 (5.1)	25.0 (5.4)	0.163
Parity			
0	43.5 (291)	43.9 (225)	
1-2	45.6 (305)	44.6 (229)	
3+	10.9 (73)	11.5 (59)	
			0.925
Time of delivery			
Night	32.3 (217)	38.5 (200)	0.026
Day	67.7 (454)	61.5 (319)	
Facility sector			
Government	94.2 (637)	91.4 (475)	
Private/Faith based	5.8 (39)	8.7 (45)	0.053
Voucher status			
No vouchers	26.7 (181)	27.0 (141)	0.915
Vouchers	73.3 (496)	73.0 (381)	

*Missing values < 2 %

^a^Based on chi-square test of association

#### Changes in observed prevalence of D & A

During examination, non-consensual care was common at baseline (61 %) and rose to even higher levels at endline (81 %) (OR 3.4; 95 % CI: 2.5–4.7) (Table 7). A significant decline in observed lack of privacy occurred between baseline and endline; however, from 34–13 % (OR 0.3; 95 % CI: 0.2–0.4). Physical aggression during late labor and delivery decreased significantly, from 3.8–0.4 % (OR 0.1, 95 % CI: 0.03–0.5), and lack of privacy, experienced by 92 % of women at baseline, declined to 79 % (AOR 0.3; 95 % CI: 0.2–0.5). Although verbal abuse decreased between baseline and endline, both during examination and delivery, neither difference was statistically significant. Women sharing beds in the postnatal ward significantly increased, from 33–to 44 % (OR 1.7, 95 % CI: 1.3–2.3). Multivariate logistic regression controlling for maternal age, parity, time of delivery, facility type and voucher status resulted in only minor changes in odds ratios for the various categories of observed D & A at baseline and endline, as illustrated in Table 8.

**Table 7:** Prevalence of recorded observations of verbal abuse and lack of privacy from partitions during examination and any aggression and privacy violations during delivery among maternity care clients whose care was observed as part of the Heshima project at baseline (2011) and endline (2014) in 13 facilities in Kenya, *n* = 1,200*

	Baseline (*n* = 677)	Endline (*n* = 523)		
	% (n)	% (n)	OR (95 % CI)^a^	*p*-value
During examination				
Non-consented care^b^	60.7 (410)	80.8 (420)	3.43 (2.52 – 4.66)	< .0001
Verbal abuse^c^	18.1 (122)	14.0 (72)	0.77 (0.55 – 1.09)	0.136
Lack of privacy^d^	33.7 (227)	12.8 (66)	0.26 (0.19 – 0.36)	<.0001
During delivery				
Physical aggression	3.8 (24)	0.4 (2)	0.11 (0.03 – 0.48)	0.003
Verbal aggression	10.8 (68)	7.1 (33)	0.68 (0.44 – 1.06)	0.091
Lack of privacy^e^	91.6 (581)	79.3 (368)	0.31 (0.20 – 0.48)	< .0001
Postpartum				
Shared bed	32.9 (210)	44.3 (198)	1.74 (1.33 – 2.28)	< .0001

*Missing values < 11 %

^a^Endline vs. baseline; facility as a random effect to account for clustering

^b^Not obtaining permission or consent before vaginal exam

^c^Use of harsh tones, shouting, or non-dignified language

^d^No partitions or partitions not closed

^e^Not covered while being moved from per-labor ward to delivery room; not covered (excluding perineal area) during delivery; partitions not closed

**Table 8:** Multivariate logistic GLMMs assessing risk factors for observed incidences of disrespect and abuse among maternity patients whose care was observed as part of the Heshima project at baseline (2011) and endline (2014) in 13 facilities in Kenya, *n* = 1,200*

	During examination			During Delivery			Postpartum
	Non-consented care^a^	Verbal abuse^b^	Lack of privacy^c^	Physical aggression	Verbal aggression	Lack of privacy^d^	Shared bed
	AOR (95 % CI)	AOR (95 % CI)	AOR (95 % CI)	AOR (95 % CI)	AOR (95 % CI)	AOR (95 % CI)	AOR (95 % CI)
Group							
Baseline	Ref^e^	Ref	Ref^e^	Ref^e^	Ref	Ref^e^	Ref^e^
Endline	3.29 (2.38 – 4.55)	0.78 (0.55 – 1.10)	0.24 (0.17 – 0.34)	0.12 (0.03 – 0.49)	0.71 (0.45 – 1.12)	0.31 (0.20 – 0.50)	1.57 (1.18 – 2.08)
Age (for each additional year)	0.99 (0.95 – 1.02) ^f^	1.00 (0.96 – 1.04)	0.97 (0.94 – 1.01)	1.01 (0.91 – 1.11)	1.03 (0.98 – 1.09)	0.98 (0.93 – 1.03)	0.97 (0.94 – 1.01)
Parity							
0	Ref	Ref	Ref	Ref	Ref	Ref	Ref
1-2	0.88 (0.63 – 1.24)	1.15 (0.78 – 1.70)	0.91 (0.64 – 1.29)	0.89 (0.36 – 2.18)	0.65 (0.40 – 1.07)	0.89 (0.53 – 1.50)	1.14 (0.83 – 1.56)
3+	1.22 (0.67 – 2.23)	1.00 (0.49 – 2.02)	1.15 (0.61 – 2.16)	0.57 (0.09 – 3.57)	0.48 (0.18 – 1.27)	1.16 (0.51 – 2.65)	1.44 (0.81 – 2.58)
Time of delivery							
Day	Ref^f^	Ref	Ref^e^	Ref	Ref	Ref^e^	Ref
Night	0.74 (0.53 – 1.02)	1.15 (0.81 – 1.64)	1.39 (1.01 – 1.92)	0.59 (0.23 – 1.50)	0.94 (0.60 – 1.49)	1.96 (1.21 – 3.18)	1.15 (0.87 – 1.52)
Facility sector							
Government	Ref	Ref	Ref	Ref	Ref	Ref	Ref
Private/Faith based	0.27 (0.04 – 2.02)	0.69 (0.12 – 3.98)	1.60 (0.28 – 9.20)	1.10 (0.12 – 10.19)	1.02 (0.24 – 4.44)	0.34 (0.01 – 8.51)	0.36 (0.03 – 4.81)
Voucher status							
No vouchers	Ref^e^	Ref	Ref	Ref^f^	Ref^f^	Ref	Ref^e^
Vouchers	7.15 (1.08 – 47.34)	2.64 (0.52 – 13.31)	1.31 (0.13 – 13.03)	4.19 (0.81 – 21.74)	3.19 (0.91 – 11.19)	2.78 (0.15 – 53.26)	13.70 (1.32 – 141.96)

*Missing values < 11 %

^a^Not obtaining permission or consent before vaginal exam

^b^Use of harsh tones, shouting, or non-dignified language

^c^No partitions or partitions not closed

^d^Not covered while being moved from per-labor ward to delivery room; not covered (excluding perineal area) during delivery; partitions not closed

^e^Statistically significant at *p* < 0.05

^f^Statistically significant at *p* < 0.10

#### Factors associated with reported D & A

Associations between socio-demographic and facility factors are shown in Table 8. Except for a facility’s voucher status, which was associated with higher risk of non-consensual care during examination (AOR 7.2; 95 % CI: 1.1–47.3) and postpartum bed sharing (AOR 13.7; 95 % CI: 1.3–142.0), the only other significant associations were for night deliveries and lack of privacy during both examination and delivery.

## Discussion

The *Heshima* study was designed to investigate the effects of an array of complementary facility and community interventions for reducing D & A during facility-based childbirth in Kenya. The intervention period was associated with an overall 7 % reduction in D & A reported by postnatal women after their discharges from maternity units, from 20–13 %, and most sub-categories of D & A declined by 40–50 %. Patient-reported declines in D & A were echoed by substantial reductions recorded for most categories of D & A during labor and delivery in the observation of patient-provider interactions.

A unique feature of our study is data reported by women themselves, as well as trained nurse and midwife researchers’ observations of labor and delivery. Research observers reported D & A with more frequency than patients in interviews, an phenomenon that is repeated elsewhere [7]. Factors contributing to this discrepancy may include women’s lower expectations for their own care, or under-reporting due to the fact patient interviews, although in private rooms, occurred within the facility compounds where women had just delivered. In other studies off-site interviews usually report considerably higher levels of D & A than interviews at the time and location of discharge [7]. Conversely, nurses and midwives may have over-reported D & A in the observation of provider-patient interaction, due to their high sensitization to D & A.

The frequency of D & A typologies varied considerably in both the interviews and observations. Rates of verbal abuse, for example, were several times higher than rates of physical abuse, in both interviews and observations. Not unexpectedly, some D & A typologies declined more than others, with the greatest decline in detention (reported in client interviews), and physical abuse (observed in provider-patient interactions). A few forms of D & A, such as non-consensual care, and abandonment, actually increased.

Although difficult to determine from the pre-and-post design, the degree of change in overall frequency for the various D & A typologies likely resulted from a combination of the intervention with other contextual factors. Values clarification and attitude training sessions, which asked providers to consider their values and attitudes exhibited while providing care, in addition to staff counseling sessions that provided opportunities for expressing their frustrations as well as support, likely contributed to greater D & A awareness. At the same time, the free maternity care inaugurated by the government during the study period resulted in increased deliveries, but unchanged staffing levels, potentially increasing risk for D & A, which may explain increased abandonment during labor and delivery as well as non-consensual care. If, in fact, greater workloads increased the stress of facility staff, it could be argued that the intervention’s measurable effects could have been even more substantial if the change in delivery policy had not occurred.

The June 2013 devolution of the health system resulted in changes in availability of frontline providers due to transfers or promotions among newly created county and sub-county health management structures. Limited corresponding recruitment of additional providers to counter internal attrition resulted in increased workload and pressures. The increased bed sharing reported in the endline observations illustrates facility challenges for expanding their infrastructures and essential supplies for their increasing populations, which was further exacerbated by the free maternity care policy.

Of particular interest is the association between occurrences of physical and verbal abuse and night shift deliveries, in both the interviews and observations, suggesting an interaction between the intervention and health system factors influencing D & A. Only about one-third of deliveries observed actually occurred during the night shift, when staffing is generally low and providers may be more likely to experience stress that may engender physical or verbal abuse. Social pressure within facilities, for adjusting to the new behavioral norms, may lessen at night because of limited patient, companion, co-worker and management pressure to adhere to these norms.

Detainment was the D & A outcome that declined the most from baseline to endline, from 8–0.8 %, also likely influenced by the changing political context and the free maternity care mandate. Although the practice of detaining women and infants on hospital premises for bill payment was never legal, our data suggest it was not rare. In the multivariate analysis, several statistically significant risk factors were associated with facility detainment, including client constituency within the poorest tercile, being unmarried, referral to the facility, and prior physical abuse. Data on complications were not available, but women of higher parities and those referred to facilities may have experienced unexpected costs associated with greater complications that those women were unable to meet, while unmarried women may have lacked financial networks for unexpected costs, and the poorest women would have been unable to pay even in the absence of complications.

This study has several significant limitations. The greatest limitation is its lack of a control group, which removes the ability to distinguish the intervention’s effect from the many contextual factors during the study period. The consistent decrease in most D & A typologies, as well as their magnitudes, in both the interview and observational studies, suggests there may have been an effect independent of concurrent temporal trends, but this study design cannot distinguish them. Future studies, with a stronger design, are needed to corroborate this study’s promising but preliminary findings.

The intervention’s relatively brief duration of 14 months in seven facilities and 20 months in the remaining six was another limitation. The D & A interventions addressed intrinsic values and behaviors that generally take considerable time to internalize, and elements addressing health system factors also require time for consistent results. It is likely the changes in D & A that were observed would have been greater if the study had continued.

Although controlled in the multivariate analyses, the baseline and endline study groups differed in several ways, which could have influenced our results. Participation rates for the baseline and endline surveys differed, with a higher percentage of women from the highest wealth tercile interviewed at endline. Women at endline were also more likely to report prior physical abuse or rape than those at baseline. The reasons for the former difference are not clear, especially after the enactment of free delivery services, which should have resulted in a higher percentage of lower tercile women in the endline survey. In addition, increased reporting of previous experiences of physical abuse or rape may have resulted from increased awareness of D & A issues following the community interventions, thus reducing its “normalization”.

The observation of provider-patient interaction enrolled only women in the early stages of labor upon facility admission, which did not represent the majority of women delivering in facilities. This limitation resulted from ethical board considerations, particularly those concerning women in active labor, who are in a much less likely position for fully informed consent to participate. It is difficult to assess whether D & A is more or less common in women presenting at facilities in later stages of labor. Although their exposure to a facility may be less, anecdotal accounts suggest that health care workers may verbally abuse them precisely because they arrive late in their labor process.

Stepped wedge designs or time series analyses would help facilitate separation of the many contextual changes in most countries’ maternal health services from the effects of interventions. More work is also needed to develop D & A measurement tools that are sensitive, specific and adaptable for different cultural contexts. Streamlined tools for measuring D & A that can be used more routinely, for quality improvement and multi-purpose surveys of health facility quality, are also needed.

## Conclusions

This study constitutes one of the first assessments of a multi-component intervention’s effects on the prevalence of D & A. Our results suggest that the implementation of such interventions within facilities and communities have the potential to reduce the occurrence of D & A and possibly to improve maternal and neonatal outcomes, although further studies with control groups or time series approaches with a longer follow up period are needed for validating these findings. This study’s iterative and learning process, an essential component of the implementation permitting careful consideration of the roles of contextual influences, will be essential for implementing similar interventions in other settings.
